# Molecular Diagnostics in Clinical Oncology

**DOI:** 10.3389/fmolb.2018.00076

**Published:** 2018-08-27

**Authors:** Anna P. Sokolenko, Evgeny N. Imyanitov

**Affiliations:** ^1^Department of Tumor Growth Biology, N.N. Petrov Institute of Oncology, St. Petersburg, Russia; ^2^Department of Medical Genetics, St. Petersburg Pediatric Medical University, St. Petersburg, Russia; ^3^Department of Oncology, I.I. Mechnikov North-Western Medical University, St. Petersburg, Russia; ^4^Department of Oncology, St. Petersburg State University, St. Petersburg, Russia

**Keywords:** carcinoma of unknown primary site, hereditary cancer syndromes, liquid biopsy, molecular diagnostics, predictive markers, review

## Abstract

There are multiple applications of molecular tests in clinical oncology. Mutation analysis is now routinely utilized for the diagnosis of hereditary cancer syndromes. Healthy carriers of cancer-predisposing mutations benefit from tight medical surveillance and various preventive interventions. Cancers caused by germ-line mutations often require significant modification of the treatment strategy. Personalized selection of cancer drugs based on the presence of actionable mutations has become an integral part of cancer therapy. Molecular tests underlie the administration of EGFR, BRAF, ALK, ROS1, PARP inhibitors as well as the use of some other cytotoxic and targeted drugs. Tumors almost always shed their fragments (single cells or their clusters, DNA, RNA, proteins) into various body fluids. So-called liquid biopsy, i.e., the analysis of circulating DNA or some other tumor-derived molecules, holds a great promise for non-invasive monitoring of cancer disease, analysis of drug-sensitizing mutations and early cancer detection. Some tumor- or tissue-specific mutations and expression markers can be efficiently utilized for the diagnosis of cancers of unknown primary origin (CUPs). Systematic cataloging of tumor molecular portraits is likely to uncover a multitude of novel medically relevant DNA- and RNA-based markers.

## Introduction

Molecular diagnostics is a part of laboratory medicine, which relies on the detection of individual biologic molecules. The potential of molecular genetic tools was initially recognized by oncohematologists, given that specific chromosomal translocations may significantly aid the diagnosis of various leukemias and lymphomas (Fey and Wainscoat, 1988). The emergence of practical applications of molecular oncology is largely attributed to the development of user-friendly methods of molecular analysis. The invention of PCR (polymerase chain reaction) led to an enormous breakthrough in clinical DNA testing: PCR-based techniques require relatively simple instrumentation and infrastructure, utilize only minute amounts of biological material and are highly compatible with clinical routine. The development of immunohistochemistry (IHC), i.e., the method allowing the visualization of specific antigen within the tissue, dates back to the mid XX century (Coons and Kaplan, 1950; Dixon and Vazquez, 1956). IHC was adapted for the clinical determination of the level of expression of estrogen receptor (ER) more than thirty years ago; this was a truly historical advance in personalized oncology, as it changed medical attitudes toward the most common oncological disease, i.e. breast cancer (BC), by tailoring endocrine therapy to a laboratory test (Coombes et al., 1987). For the time being, some conventional protein-targeted tests, e.g., IHC or determination of tumor-specific serum markers (PSA, CA-125, etc.), are rarely discussed in the framework of molecular diagnostics. The latter term is usually applied to DNA- or RNA-based assays as well as to some modern sophisticated proteomic technologies.

**Figure 1:**
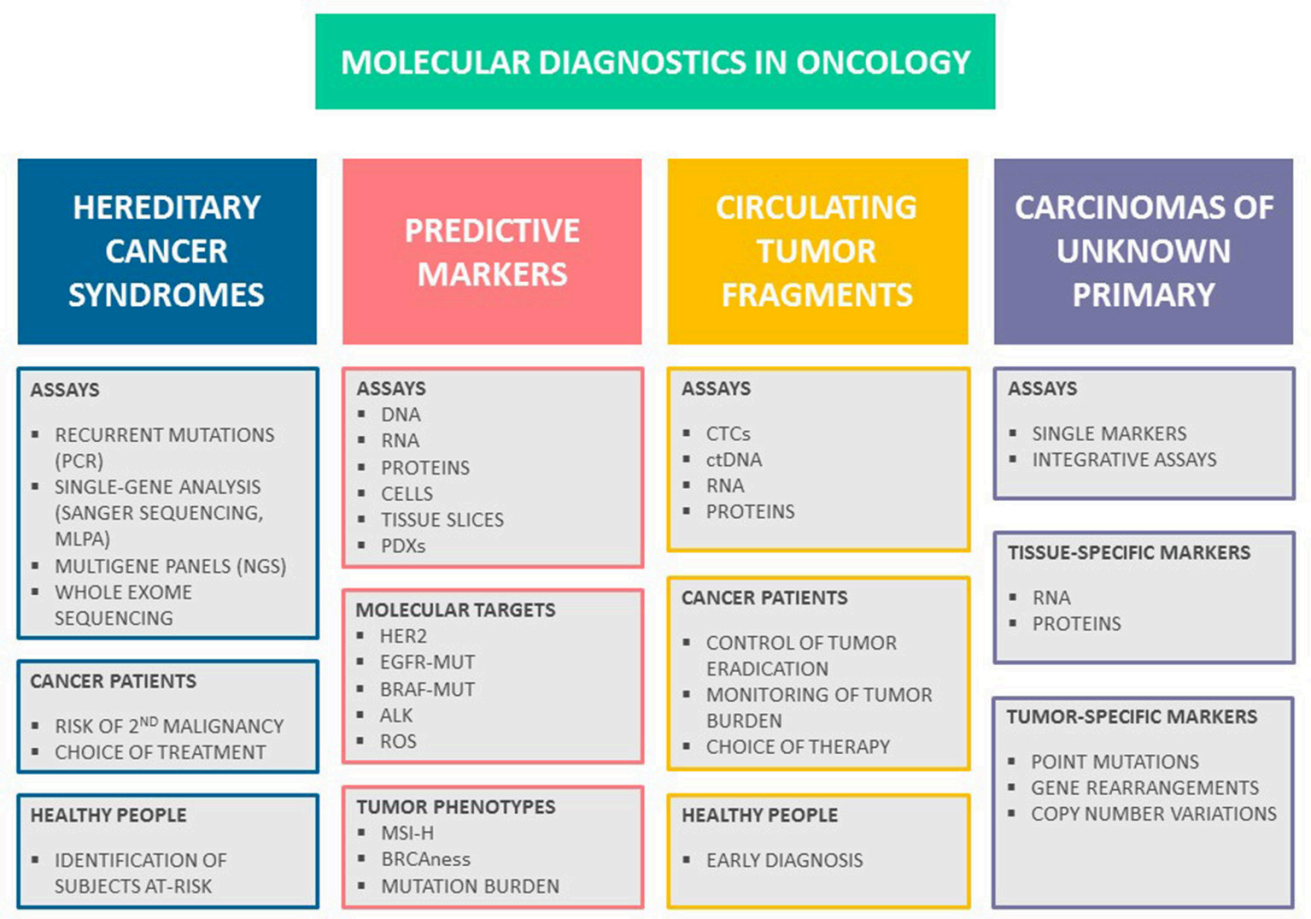
Molecular diagnostics in oncology. There are several major avenues in cancer medicine, which utilize molecular-based assays. Testing for hereditary cancer syndromes is now routinely used both for identification of persons at-risk and for personalization of systemic treatment. There is a number of predictive tests involving either the analysis of individual drug targets or identification of specific tumor phenotypes, which aid the choice of anticancer drugs. Monitoring of malignant disease can be achieved through molecularly-driven detection of residual tumor fragments; it is anticipated that liquid biopsy will serve as an instrument for early cancer diagnosis and screening in the future. Recent developments in the mutation testing and RNA analysis offer novel tools for diagnosis of cancers of unknown primary site.

There are two avenues where molecular tests have become a part of standard patient management (Figure 1). First, identification of subjects with hereditary cancers is now a daily practice in clinical oncology. Second, there is a number of tests, which help to select the most effective treatment based on molecular characteristics of tumor tissues or some other biologic parameters of malignant disease. There are some additional applications, which remain in the developmental stage. In particular, some of modern molecule-oriented techniques virtually do not have a sensitivity limit, therefore there are intensive efforts to apply these tests for monitoring of residual cancer disease and early tumor detection. In addition, DNA and RNA assays may help to differentiate between tumors of distinct histologic origin, which is suitable for diagnosis of cancers of unknown primary site (CUPs). This review is focused on the recent achievements in molecular diagnostics of cancer; literature search criteria utilized for the preparation of this articles are given in Supplementary Material.

## Hereditary cancer syndromes

Hereditary cancer syndromes compose a group of genetic defects, which render highly significant elevation of cancer risk; importantly, this risk is more or less organ-specific, which allows to arrange meaningful diagnostic and preventive interventions for germ-line mutation carriers. Hereditary cancers are by far more common than “classical” genetic diseases: for example, population frequency of breast or ovarian cancers associated with *BRCA1/2* gene defects approaches to 1:500 and even reaches 1:50 in some founder populations (Satagopan et al., 2001; Risch et al., 2006; Foulkes et al., 2016), while the most known non-cancer hereditary syndromes, e.g., cystic fibrosis or phenylketonuria, are less frequent at least by an order of magnitude (Strausbaugh and Davis, 2007; Berry et al., 2013). Hereditary cancers may have peculiar clinical appearance, such as early onset, presence of multiple neoplasms and preference toward particular histological pattern.

The genetic diagnosis of hereditary cancer became possible by the identification of germ-line mutations in corresponding genes. The discovery of retinoblastoma gene was a pioneering event in this field, as it provided a tool for the management of families with this rare pediatric tumor of the eye (Horsthemke et al., 1987; Bookstein et al., 1988). Malkin et al. (1990) later described a genetic cause of another rare cancer predisposition disease, so-called Li-Fraumeni syndrome: it turned out that this severe multiorgan tumor syndrome is caused by then already well-known suppressor gene *p53*. Soon afterwards, Nishisho et al. (1991) and Kinzler et al. (1991) discovered the genetic basis of familial adenomatous polyposis (FAP), i.e., germ-line mutations in *APC* (adenomatous polyposis coli) gene. First genes for hereditary non-polyposis colorectal cancer were identified within the years 1993–1994 (Fishel et al., 1993; Bronner et al., 1994). However, the long-awaited discovery of genes for breast-ovarian cancer syndrome, *BRCA1* and *BRCA2* (Miki et al., 1994; Wooster et al., 1994), received even more attention from the media, probably due to high prevalence of this disease.

Germ-line mutations in *BRCA1* and *BRCA2* genes are associated with ~60–90% probability of developing breast cancer and 40–60% life-time risk of ovarian carcinoma (Antoniou et al., 2003). *BRCA1/2* mutations are responsible for 5–8% of breast and 10–20% ovarian cancer morbidity. *BRCA1/2* heterozygosity also contributes to some instances of pancreatic, prostate and gastric cancers, although these associations are described in a less systematic way as compared to the neoplasms of female reproductive tract (Moiseyenko et al., 2013; Cavanagh and Rogers, 2015). Cancers arising in *BRCA1/2* mutation carriers are often of high grade and more chromosomally unstable than their sporadic counterparts (Eerola et al., 2005; Alexandrov et al., 2013). Medical surveillance for *BRCA1/2* heterozygotes reduces the risk of dying from breast cancer, however it is unlikely to affect the ovarian cancer related mortality (van der Velde et al., 2009; Møller et al., 2013). Given the limitations in early diagnosis and treatment of *BRCA1/2*-driven breast and ovarian carcinomas, prophylactic surgery is considered to be a standard option for clinical management of *BRCA1/2* mutation carriers (Fatouros et al., 2008).

A number of novel BC-predisposing genes have been identified in the past. *PALB2* is among the most validated ones, being characterized by noticeable contribution in BC incidence and relatively high penetrance (Antoniou et al., 2014). There is a couple of middle-penetrance genes (*CHEK2, ATM, NBN, BLM*, etc.), which were shown to increase the risk of the disease, but to a lesser extent than *BRCA1* or *BRCA2* mutations (Bogdanova et al., 2013).

Hereditary non-polyposis colorectal cancer (HNPCC) syndrome (also known as Lynch syndrome) is caused by germ-line mutations in *MLH1, MSH2, MSH6, PMS2*, and *EPCAM* genes (Lynch et al., 2015). The distinct feature of tumors associated with this syndrome is a so-called high-level microsatellite instability (MSI-H), which is caused by inactivation of DNA mismatch repair (MMR) genes. Besides colorectal cancer, female carriers of genetic defects in the above genes are at very high risk of developing endometrial cancer. In addition, Lynch syndrome is associated with increased risk of neoplasms of the stomach, ovary, bladder, etc. Cancers arising in patients with HNPCC have somewhat better prognosis as compared to sporadic malignancies, probably due to excessive mutation burden, and consequently, high level of antigenicity. This favorable disease course explains excellent results of medical surveillance in healthy subjects belonging to hereditary colorectal cancer families, with no tumor-related deaths observed in compliant individuals (Järvinen et al., 2000).

The procedure of molecular diagnosis of hereditary cancer is affected by many factors. There is a number of relatively simple laboratory tests, which can be applied without major limitations even to persons with minor suspicion for familial cancer syndrome. For example, some countries and/or ethnic groups (Ashkenazi Jews, Icelanders, Eastern Slavs) are characterized by a pronounced founder effect for *BRCA1/2* mutations, where the majority of *BRCA1/2* gene lesions can be explained by the persistence of just a few alleles (Abbott, 2000; Ferla et al., 2007; Kurian, 2010; Foulkes et al., 2016). Therefore, it is advisable to use these cheap PCR tests virtually for every patient with breast or ovarian cancer, or even for population screening in the respective ethnic communities. In contrast to *BRCA1*, recurrent pathogenic variants in *APC* gene are caused not by a founder effect, but by the existence of the mutation hotspots in the codons 1309 and 1062 (Yanus et al., 2018). In some instances, the phenotypic appearance of the tumor may guide subsequent diagnostic procedures. For example, microsatellite instability testing, which can be achieved either by IHC or by PCR, is a reliable selection test for the patients carrying germ-line mutations in HNPCC-related genes (Lynch et al., 2015; Buza et al., 2016; Gelsomino et al., 2016). Triple-negative breast carcinomas are candidates for *BRCA1* gene testing (Engel et al., 2018). Furthermore, comprehensive analysis for *BRCA1/2* germ-line mutations is recommended to all patients with high-grade serous ovarian cancer (Neff et al., 2017). The diagnosis of medullary thyroid carcinoma calls for investigation of germ-line status of *RET* oncogene (Figlioli et al., 2013).

Comprehensive diagnosis of hereditary cancer syndromes presents a challenge even in the postgenomic era. Most of known familial cancer types are represented by phenocopies, i.e., several distinct genes may cause similar or overlapping phenotypic manifestation. For example, the analysis of mutations related to breast cancer risk would include *BRCA1, BRCA2, PALB2, TP53, CHEK2, ATM, NBS/NBN, BLM, PTEN, MRE11, BRIP1, BARD1, RAD50, RAD51C, RAD51D, RECQL, FANCC, FANCM* and perhaps some other genes (Sokolenko et al., 2012; Thompson et al., 2012; Kiiski et al., 2014; Kurian et al., 2014; Cybulski et al., 2015; Easton et al., 2015). Similarly, genetic testing for HNPCC requires the analysis of *MLH1, MSH2, MSH6, PMS2*, and *EPCAM* coding sequences, while the panel for diagnosis of colon polyposis would involve *APC, MUTYH, NTHL1, POLE, POLD1, SMAD4, BMPR1A, STK11*, and *MSH3* (Weren et al., 2015; Adam et al., 2016; Bellido et al., 2016; Kanth et al., 2017). Valid conclusions on the lack of causative mutation cannot be made solely on the basis of Sanger sequencing; it is often somehow overlooked, that many germ-line mutations are represented by large gene rearrangements (LGRs), which require distinct diagnostic platforms, e.g., multiplex ligation-dependent probe amplification (MLPA) or droplet digital PCR (ddPCR) (Ewald et al., 2009; Sluiter and van Rensburg, 2011; Preobrazhenskaya et al., 2017). The invention of next-generation sequencing (NGS) dramatically facilitated the access to multigene analysis. There is a number of NGS panels, which provide information on mutation status of dozens of cancer-causing genes. They are characterized by excellent technical performance, demonstrating virtually null rate of false results (Lincoln et al., 2015). However, most of available diagnostic panels mix together genes with well-established medical significance and gene-candidates with poorly proven disease-predisposing role (Easton et al., 2015; Sokolenko and Imyanitov, 2017). Therefore, significant clinical genetic expertise needs to be involved in interpreting high-throughput DNA tests.

There are major limitations in the diagnostics of hereditary tumor syndromes. In contrast to non-cancer genetic diseases, where significant knowledge is accumulated with regard to pathogenic role of aminoacid substitutions, the spectrum of tumor-predisposing alleles is almost entirely represented by protein-truncating variants (Sokolenko et al., 2015). It is beyond any reasonable doubt that many missense mutations, which are currently classified as “variants of unknown significance,” are actually disease-predisposing. For the time being, there is no efficient pipeline, which would allow to classify rare aminoacid substitutions for benign and disease-predisposing mutations.

Most of diseases known in medical genetics are autosomal-recessive, i.e., the affected subjects carry biallelic abnormalities in a certain gene, while their parents are asymptomatic heterozygous mutation carriers. In contrast, virtually all known cancer syndromes have a dominant mechanism of inheritance, i.e., most of the affected subjects have heterozygous genotype for the involved genes. This difference cannot be attributed to biologic reasons, but instead is explained by the mode of discovery of the above diseases. Classical genetic maladies are exceptionally rare disorders, which are characterized by authentic disease presentation and usually discovered by observing a few cases of the same orphan disease in the same family and/or neighborhood. In contrast, hereditary cancers are not at all phenotypically authentic but masked by the excess of sporadic phenocopies. As a result, the most known cancer syndromes were discovered by the analysis of extensive multi-generation pedigrees with an outstandingly high occurrence of particular cancer type. Rapidly increasing accessibility of comprehensive genomic analysis is very likely to reveal many examples of recessive inheritance of cancer predisposition. Biallelic tumor-predisposing mutations are probably responsible for a significant share of early-onset and multiple cancers, especially in those subjects, who do not report a family history of the disease or exposure to environmental hazards (Kuligina et al., 2013).

It is important to realize that most of (cancer) genetic studies were carried out in North America and Western Europe, therefore they reflect the mutation burden in subjects of European descent. It is very likely that people of other ethnicities and races inherited totally another pattern of pathogenic mutations from their founders. This is well exemplified by the molecular epidemiology of *BRCA1* and *BRCA2* mutations, which show significant global variations with regard to contribution in regional cancer incidence as well as to mutation spectrum (Kurian, 2010). Only a minor part of the heritability of cancer risk has been dissected so far, and forthcoming whole exome sequencing studies are expected to significantly increase the number of known hereditary cancer genes. The existence of medically relevant interethnic genetic variations needs to be considered while planning these activities (Sokolenko et al., 2015).

The most known hereditary cancer syndromes are listed in Table 1.

**Table 1:** Hereditary cancer syndromes: selected examples.

**Syndrome**	**Gene**	**Tumors**	**Comments**	**References**
Hereditary breast-ovarian cancer (HBOC)	*BRCA1, BRCA2, PALB2*	Breast, ovarian, pancreatic, prostate, gastric cancer	Tumors are deficient for double-strand break DNA repair by homologous recombination	Miki et al., 1994; Moiseyenko et al., 2013; Antoniou et al., 2014; Cavanagh and Rogers, 2015
Hereditary breast cancer: novel and/or moderately penetrant genes	*CHEK2, ATM, BARD1, BLM, BRIP1, NBS/NBN, MRE11, RAD50, RAD51C, RAD51D, FANCC, FANCM*	Breast cancer		Sokolenko et al., 2012; Thompson et al., 2012; Bogdanova et al., 2013; Kiiski et al., 2014; Cybulski et al., 2015; Easton et al., 2015
Lynch syndrome, or hereditary nonpolyposis colorectal cancer (HNPCC)	*MLH1, MSH2, MSH6, PMS2, EPCAM*	Colon, endometrial, breast, urothelial, small intestine, gastric cancer	High-level microsatellite instability in tumors	Lynch et al., 2015
Hereditary colorectal cancer	*POLE, POLD1*	Polyposis, colorectal cancer	Very high mutation burden in tumors	Bellido et al., 2016
Familial adenomatous polyposis (FAP)	*APC*	Multiple (>100) colonic adenomas, desmoid tumors, colorectal cancer		Fishel et al., 1993
MUTYH-associated polyposis (MAP)	*MUTYH*	Moderate number of colonic adenomas, colorectal cancer	Autosomal-recessive inheritance	Kanth et al., 2017
NTHL1-associated polyposis (NAP)	*NTHL1*	Polyposis, colorectal cancer	Autosomal-recessive inheritance	Weren et al., 2015
Juvenile polyposis	*SMAD4, BMPR1A*	Colorectal polyps, colorectal cancer, other gastrointestinal cancers		Kanth et al., 2017
Peutz-Jeghers syndrome	*STK11*	Hamartomatous polyps, gastrointestinal cancers		Kanth et al., 2017
Hereditary diffuse gastric cancer	*CDH1*	Gastric cancer		Oliveira et al., 2009
Li-Fraumeni syndrome	*TP53*	Soft tissue sarcomas, breast cancer, brain tumors, adrenal gland cancer		Ruijs et al., 2010
Multiple endocrine neoplasia type 1	*MEN1*	Parathyroid, pituitary gland, gastroenteropancreatic tumors		Norton et al., 2015
Multiple endocrine neoplasia type 2	*RET*	Medullary thyroid carcinoma, pheochromocytoma	Gain-of-function germ-line mutations	Norton et al., 2015
Von Hippel-Lindau disease	*VHL*	Clear cell renal cell carcinoma, hemangioblastomas of the brain, other tumors		Latif et al., 1993; Friedrich, 1999
Cowden syndrome	*PTEN*	Multiple hamartomas, breast cancer, thyroid cancer		Kanth et al., 2017
Familial retinoblastoma	*RB1*	Retinoblastoma		Lohmann, 1999
Familial melanoma	*CDKN2A, CDK4, TERT, POT1*	Melanoma		FitzGerald et al., 1996; Horn et al., 2013; Robles-Espinoza et al., 2014

*Comprehensive cataloging of hereditary cancer syndromes is beyond the scope of this review. Some of the described tumor diseases have a relatively high prevalence, e.g., hereditary breast-ovarian cancer syndrome or Lynch syndrome. Other examples represent orphan diseases; however, some of them, e.g., familial retinoblastoma or Li-Fraumeni syndrome, are widely known in medical and research community because their identification resulted in significant breakthrough in basic understanding of cancer pathogenesis. This table does not include severe multiorgan maladies, in which cancer serves only as part of clinical manifestation, i.e., some primary immune deficiencies, DNA repair abnormalities etc. The list of cancer syndromes is constantly expanding due to discovery of novel causative genes (Sokolenko et al., 2015). The catalog of cancer syndromes can be found in the OMIM database (https://www.ncbi.nlm.nih.gov/omim/)*.

## Molecular markers for the choice of cancer therapy

First examples of the use of predictive markers in oncology were related to breast cancer research (Engelsman, 1974; Jensen, 1975). The analysis of expression of estrogen receptor in breast cancer tissues demonstrated that only ER-positive BC benefit from ovariectomy or other types of estrogen ablation. First ER expression assays required sophisticated biochemical analysis of fresh tumor tissue and therefore were available only in advanced medical centers (McGuire, 1973; Heuson et al., 1977). The invention of ER IHC analysis made this test accessible worldwide (Coombes et al., 1987). Nowadays, determination of the status of estrogen and progesterone receptors is a mandatory part of BC diagnosis, which guides the use of endocrine therapy. There is a continuous adjustment of IHC-based hormone receptor assays to the clinical requirements of BC management (Fujii et al., 2017).

The discovery of *HER2* amplification and overexpression in breast cancer eventually led to the development of several HER2-targeted therapies, with trastuzumab being the first-in-class drug. From the very beginning, the administration of trastuzumab was tailored to women, whose tumors showed clear evidence for *HER2* gene activation. The analysis of *HER2* status is now routinely utilized in the management of breast and stomach cancer (Sauter et al., 2009; Bartley et al., 2017). In addition, there are some other examples of *HER2*-driven malignancies, which demonstrate a pronounced benefit from HER2 inhibition (Sartore-Bianchi et al., 2016).

Epidermal growth factor receptor (EGFR) inhibitors entered clinical trials in the beginning of the last decade. It was assumed that virtually all types of epithelial malignancies are characterized by some involvement of EGFR activation, therefore EGFR-directed therapies were expected to be efficient in a wide spectrum of cancers (Nicholson et al., 2001; Baselga, 2002). First trials on a small-molecule EGFR tyrosine kinase inhibitor (TKI), gefitinib, involved heavily pretreated patients with the lack of available standard treatment options. Impressively, 4 out of 16 lung cancer (LC) patients included in the phase I study demonstrated the response to the drug (Ranson et al., 2002). These observations were confirmed in subsequent phase II studies, where some proportion of LC patients experienced dramatic tumor reduction upon gefitinib therapy (Fukuoka et al., 2003). It remained unknown, why this drug rendered clear and immediate benefit to some small subset of LC patients while being overtly ineffective in the majority of LC cases. In the year 2004, three research groups independently reported the results of *EGFR* gene sequencing in tumors obtained from EGFR TKI responders and non-responders. It turned out that cancers from virtually all responders carried by then unknown intragenic mutation in *EGFR* gene, while non-responders were characterized by the wild-type *EGFR* sequence (Lynch et al., 2004; Paez et al., 2004; Pao et al., 2004). This discovery opened an era of mutation-specific drugs, and *EGFR* mutation testing is now a standard diagnostic procedure in LC management.

The predictive role of *ALK* translocations was also revealed due to a chance. Crizotinib was initially invented as a MET inhibitor, and its activity against ALK kinase was not considered to be clinically important at the time of drug development (Christensen et al., 2007; Shaw and Solomon, 2011). Early crizotinib clinical trials coincided with the discovery of *ALK* translocations in lung cancer (Soda et al., 2007; Kwak et al., 2009). The analysis of drug responders revealed that the presence of *ALK* fusions was the major prerequisite for the drug efficacy. Subsequent studies demonstrated that a similar kinase, *ROS1*, is also recurrently rearranged in LC, and *ROS1*-driven tumors represent another category of crizotinib-responsive cancers (Shaw et al., 2014). *ALK* and *ROS1* rearrangements are now routinely tested in lung adenocarcinomas. In addition, *ALK* and *ROS1* fusions are highly characteristic for inflammatory myofibroblastic tumors (IMT) (Yamamoto et al., 2016). The efficacy of crizotinib in *ALK*-rearranged IMT has already been confirmed in a recent clinical trial (Schöffski et al., 2018).

In contrast to *EGFR* and *ALK, BRAF* mutations were identified through intentional mutation screening of protein kinases (Davies et al., 2002). The inhibitors of mutated BRAF, vemurafenib, and dabrafenib, made a breakthrough in the treatment of *BRAF*-driven melanomas, especially when given in combination with MEK inhibitors (Ugurel et al., 2017). In addition, therapeutic inhibition of RAF/MEK signaling module is a treatment of choice for lung tumors carrying *BRAF* V600E mutation (Leonetti et al., 2018). There is a number of other tumor entities, which demonstrate modest or high occurrence of *BRAF* mutations (Hyman et al., 2015).

Clinical trials on colorectal cancer (CRC) involving anti-EGFR antibodies, cetuximab and panitumumab, convincingly demonstrated that these drugs do not render benefit in tumors carrying *KRAS* or *NRAS* mutation. RAS proteins are the members of EGFR pathway, being located downstream to the receptor. There are two categories of colorectal carcinomas. More than a half of CRCs carry mutation in either *KRAS* or *NRAS* gene, therefore the persistent activation of EGFR pathway is independent from the status of the receptor. However, EGFR activation is often a driving event in those CRCs, which are wild-type for *KRAS, NRAS*, or *BRAF* genes (Siddiqui and Piperdi, 2010; Waring et al., 2016; van Brummelen et al., 2017). *KRAS* and *NRAS* testing is now a mandatory procedure for all CRC patients considered for cetuximab and panitumumab treatment, as these drugs have virtually null efficacy in patients with mutation but provide reasonable benefit in the wild-type cases. It is of notice that the absence of mutations in *RAS/RAF* genes does not guarantee the response to anti-EGFR antibodies, as a significant portion of tumors without mutations in the above genes remain insensitive to EGFR-directed therapy (Custodio and Feliu, 2013).

Cancers arising in carriers of *BRCA1/2* germ-line mutation demonstrate an elegant therapeutic window (Iyevleva and Imyanitov, 2016). *BRCA1* and *BRCA2* genes are involved in the double-strand break DNA repair by homologous recombination. *BRCA1/2* heterozygosity is tolerated by the cells due to the activity of the remaining *BRCA1/2* allele. The pathogenesis of *BRCA1/2*-driven cancers usually involves somatic inactivation of the wild-type copy of the involved gene; therefore, malignant cells are characterized by tumor-selective deficiency of DNA repair. This makes tumor cells vulnerable to some cytotoxic drugs (platinum compounds, mitomycin C, etc.) as well as inhibitors of poly (ADP-ribose) polymerase (PARPi). In accordance with this mechanism, somatic loss of the normal *BRCA1/2* allele correlates with drug sensitivity of tumors arising in *BRCA1/2* mutation carriers (Maxwell et al., 2017). Furthermore, the development of resistance to cisplatin or PARPi involves restoration of BRCA1/2 function, achieved either by the second mutation in the affected copy of the gene or selection of pre-existing BRCA1-proficient tumor cells (Lord and Ashworth, 2013; Sokolenko et al., 2017).

High-level microsatellite instability is a phenotypic feature of cancers, which develop due to deficiency of DNA mismatch repair. MSI-H is manifested by multiple changes in the length of short repetitive DNA sequences, so-called microsatellites. Both hereditary and sporadic MMR-deficient tumors are characterized by dramatic increase in the number of coding mutations; therefore, they carry a high amount of neoantigens. Accordingly, MSI-H neoplasms are distinguished by a particularly good response to immune checkpoint inhibitors (Le et al., 2017). The discovery of this association led to the first precedent, where the drug approval was not bound to any particular tumor type, but instead relied solely on a molecular marker: indeed, an anti-PD1 therapeutic antibody, pembrolizumab, is now recommended as a standard treatment for the tumors with deficient mismatch repair (Prasad et al., 2018). While MSI-H phenotype was known since the beginning of the 1990s, the identification of hypermutated tumors driven by mutations in DNA polymerases *POLE* and *POLD1* is a relatively recent discovery (Briggs and Tomlinson, 2013; Palles et al., 2013). Similarly to MMR-deficient malignancies, these carcinomas are also characterized by a relatively favorable prognosis and responsiveness to the modulators of immune response (Mehnert et al., 2016; Santin et al., 2016; Nebot-Bral et al., 2017). Tumors with MSI-H or mutations in *POLE* or *POLD1* genes, which carry an extraordinarily excessive number of somatic genetic events, compose only a small proportion of human neoplasms. The increase of mutation burden in smoking- or ultraviolet-induced cancers is not as high; however, it is still clinically significant to predict good response to immune therapy. While the analysis of MSI-H status or inactivation of *POLE* or *POLD1* genes relies on relatively straightforward laboratory tests, the determination of tumor mutation burden in, e.g., lung cancers or melanomas, is more complicated: it is based on the whole exome sequencing, deals with continuous variable and does not have yet established thresholds for clinical decisions (Rizvi et al., 2015; Van Allen et al., 2015). Mutations in *SERPINB3* and *SERPINB4* genes were shown to correlate with overall mutation load in melanoma and therefore may potentially serve as suitable predictive markers (Riaz et al., 2016). Genomic instability caused by *BRCA1/2* gene inactivation may also result in tumor responsiveness to immune therapy (Nolan et al., 2017).

For the time being, the number of established predictive markers approaches several dozens or even hundreds, depending on criteria used for this definition. Very most of these markers are highly organ-specific: for example, *EGFR* mutations occur mainly in lung adenocarcinomas, while their incidence in other cancer types is limited to anecdotal reports (Lee et al., 2005; Iyevleva et al., 2009). Consequently, single-gene tests are usually applied only to those malignancies, where the probability of detecting corresponding targetable alteration is at least 2–5%; for example, *EGFR* testing is a standard diagnostic procedure for non-small cell lung cancer, but is actually never used for other tumor entities. This approach, although being practical, is likely to miss a significant number of potentially druggable neoplasms. To overcome this problem, some advanced cancer centers began to routinely utilize multigene platforms, which are based on the next generation sequencing analysis and include almost all known genetic loci relevant for the choice of cancer drugs. This approach helps to find promising drug-gene matches in ~1 out of 10 cancer patients (Pauli et al., 2017; Zehir et al., 2017).

The main issue in this tissue-agnostic approach is whether a particular mutation-specific drug would work in any cellular context, or instead, would remain effective only in those tumors, where the presence of specific targets is combined with the favorable architecture of relevant signaling pathways. Both possibilities were confirmed by clinical experience. For example, inhibitors of mutated *BRAF*, which were initially evaluated for the treatment of melanoma, demonstrated clinically significant efficacy in some hematological malignancies, pediatric tumors, clear cell sarcomas, but turned out to be virtually useless when applied as single-agents to gastrointestinal cancers (Hyman et al., 2015; Protsenko et al., 2015; Dietrich et al., 2016). The latter limitation is caused by the feedback activation of EGFR pathway and can be overcome by concurrent administration of EGFR inhibitors (Prahallad et al., 2012; Yaeger et al., 2015; Silkin et al., 2016). There is a number of clinical trials, which match patients to the drugs according to the presence of potentially targetable genetic lesions (Le Tourneau et al., 2015; Wheler et al., 2016; Massard et al., 2017). By their nature, these trials involve very diverse populations of heavily pretreated patients and utilize a multitude of markers with varying level of predictive significance. Le Tourneau et al. (2015) presented the results of the SHIVA trial and concluded that the wide-scale tissue-agnostic use of targeted agents outside their validated clinical indications has limited chances to deliver significant benefit to the patients, even if the latter present with potentially relevant cancer markers. Wheler et al. (2016) utilized NGS-based comprehensive genomic profiling for a spectrum of cancer types and also observed border-line if any benefit from genetically tailored drugs. More encouraging results were obtained in the MOSCATO 01 trial (Massard et al., 2017). It enrolled 1,035 patients, of whom 948 were successfully biopsied. The molecular profiles were obtained for 843 patients, with 411 tumors carrying potentially actionable molecular alterations. 199 patients were treated by molecularly tailored therapies. Importantly, in 63/193 (33%) evaluable patients the progression-free survival (PFS) on genetically matched therapy evidently exceeded the PFS obtained on the prior line of systemic treatment. This trial convincingly demonstrated, that some patients may indeed benefit from marker-based therapy administration in the tissue-agnostic setting, although the overall proportion of these instances is relatively small (~7% the MOSCATO 01 trial). Still, tissue-agnostic trials produce much more interpretable results if they focus on a single drug and a well-defined molecular target (Hyman et al., 2015; Gambacorti-Passerini et al., 2018). Overall, the development of tissue-agnostic marker-based indications for targeted drugs is getting more and more common (Garber, 2018).

There is a number of biological approaches, which aim at direct determination of the spectrum of tumor drug sensitivity. The establishing of the tumor cell lines is not a practical approach at the moment: only a minor part of naturally occurring tumors can be converted to viable cell cultures, and the properties of the obtained cell clones do not necessarily reflect the biological features of the original tumors (Kreahling and Altiok, 2015; Izumchenko et al., 2017; Pauli et al., 2017). Transplantation of the tumors to immune-deficient mice could be advantageous in terms of the success rate and mimicking the native physiological conditions for cancer growth. Patient-derived xenografts (PDXs) are being increasingly utilized in drug trials as well as in patient management undertaken in advanced cancer centers (Evans et al., 2017; Pauli et al., 2017; Yao et al., 2017). Clinical studies demonstrate that mouse PDXs mirror therapeutic responses observed in patients with a remarkable level of accuracy (Izumchenko et al., 2017). While murine experiments are expensive and time-consuming, there are efforts to establish short-term PDXs in zebrafish models (Fior et al., 2017). Lack of proper immune context and absence of human tissue environment are considered as critical limitations of PDX-based assays (Cassidy et al., 2015). Further, although PDXs are characterized by relatively good preservation of original tumor molecular portraits (Izumchenko et al., 2017), the successful engraftment of neoplasms in mice nevertheless involves some additional genetic events (Ben-David et al., 2017). Animal experiments demonstrate that topical intratumoral microinjection of cancer drugs followed by microscopic analysis of drug-exposed tumor areas may be a promising predictive test (Jonas et al., 2015; Klinghoffer et al., 2015).

It is essential to recognize that all current predictive tests ignore the issue of intratumoral heterogeneity. Multiple evidences suggest that tumors consist of distinct populations of transformed cells, which are characterized by substantial subclonal genetic diversity and epigenetic plasticity. Even if the treatment is apparently effective and results in the shrinkage of the gross tumor mass, it is unlikely to eliminate all cancer cells and may even promote the expansion of drug-resistant clones (Amirouchene-Angelozzi et al., 2017; McGranahan and Swanton, 2017; Sokolenko et al., 2017) These properties of malignant tumors explain, why metastatic disease remains largely incurable even for well-druggable cancer types.

The development of cancer drugs and corresponding predictive markers currently focuses mainly on tumor molecular portraits. A thorough consideration of other relevant factors may provide some unexpected opportunities. For example, cancers arising in visceral organs were long considered to be sterile, similarly to normal tissues. Recent data indicate that some tumors may be colonized by specific microorganisms, and these bacteria may participate in drug metabolism and contribute to the treatment response (Bullman et al., 2017; Geller et al., 2017). For example, pancreatic carcinomas often contain viable *Gammaproteobacteria*, which are capable to metabolize gemcitabine into an inactive compound (Geller et al., 2017). There are convincing evidences demonstrating that composition of gut microbiome influences the interaction between tumor and systemic therapy. For example, the intestinal microbe *Akkermansia muciniphila* was shown to mediate the efficacy of immune checkpoint modulators (Routy et al., 2018). The outcome of immune therapy may also critically depend on the patient age and HLA genotype (Champiat et al., 2017; Chowell et al., 2018). Surgical intervention may trigger the growth of dormant cancer cells by inducing the systemic inflammatory response and therefore significantly affect the probability of cancer relapse (Krall et al., 2018).

Examples of predictive markers are given in Table 2.

**Table 2:** Predictive molecular tests: selected examples.

**Drugs**	**Markers**	**References**
Tamoxifen and aromatase inhibitors	Estrogen receptor expression	Fujii et al., 2017
HER2-directed therapies	*HER2* amplification and overexpression	Sartore-Bianchi et al., 2016
ALK/ROS1 inhibitors	*ALK* and *ROS1* rearrangements	Soda et al., 2007; Kwak et al., 2009; Shaw et al., 2014
EGFR-directed therapies (sensitivity)	*EGFR* mutations	Lynch et al., 2004; Paez et al., 2004; Pao et al., 2004
EGFR-directed therapies (resistance)	*KRAS/NRAS/BRAF* mutations	Siddiqui and Piperdi, 2010; Waring et al., 2016; van Brummelen et al., 2017
PARP inhibitors	*BRCA1/2* mutations, BRCAness	Iyevleva and Imyanitov, 2016; Lord and Ashworth, 2016
Platinum compounds, mitomycin C	*BRCA1/2* mutations, BRCAness	Iyevleva and Imyanitov, 2016; Lord and Ashworth, 2016
PD1-directed therapies	High PD-L1 expression	Kumar et al., 2017
Immune checkpoint inhibitors	Tumor mutation burden	Rizvi et al., 2015
BRAF inhibitors	*BRAF* mutations	Ugurel et al., 2017; Cheng et al., 2018
mTOR inhibitors	*TSC1/2* mutations, *MTOR* mutations	Kwiatkowski et al., 2016
MET inhibitors	*MET* exon 14 skipping	Pilotto et al., 2017

## Liquid biopsy

Tumors almost always shed some amount of their fragments into peritumoral space. These fragments may be represented by single malignant cells or their clusters as well as by various proteins, nucleic acids, small molecules, etc. Consequently, these entities can be collected in various body fluids (serum, saliva, urine, etc.) and serve as tumor markers.

Serum protein markers are the most established tools for cancer diagnosis (Duffy, 2013). For example, PSA (prostate-specific antigen), CA-125 (cancer antigen 125) and CEA (carcinoembryonic antigen) are widely used for the diagnosis and management of prostate, ovarian and gastrointestinal cancers, respectively. Measurement of serum antigens allows to discriminate between various types of malignancies at the first suspicion for cancer disease and thus guide imaging analysis, endoscopic examination and other diagnostic procedures. Monitoring of the level of tumor-specific protein markers in patients with the established diagnosis of cancer permits the disease monitoring, e.g., evaluation of the efficacy of the treatment or detection of tumor relapse. PSA is used in some countries for prostate cancer screening. Protein markers, being the only type of liquid biopsy widely incorporated in routine clinical practice, have significant limitations in terms of sensitivity and specificity. It is expected that the revolution in molecular genetic research will dramatically improve the performance of fluid-based assays.

Indeed, DNA markers may have significant advantages as compared to proteins. Several methods of genetic analysis, particularly PCR and NGS, are potentially capable to detect a single molecule of tumor-specific DNA even in the presence of an excess of normal nucleic acids. Furthermore, while the majority of known protein markers are not truly tumor-specific but rather tissue-specific, DNA tests usually rely on cancer-driving mutations and therefore are less prone to false-positive results. Modern methods of liquid biopsy are not limited to the analysis of tumor-free DNA. For example, some experimental approaches utilize the detection of circulating microRNAs owing to their relatively good stability in body fluids. In addition, a number of investigational diagnostic procedures rely on the isolation and analysis of circulating tumor cells (Antonarakis et al., 2014; Dasgupta et al., 2017; Siravegna et al., 2017; Bidard et al., 2018).

While discussing the perspectives for the liquid biopsy, it is critically important to recognize that its actual relevance may significantly depend on particular clinical context. For example, virtually all current diagnostic standards for cancer patients require mandatory biopsy of the tumor lump followed by morphological validation of the presence of malignant disease. Therefore, if one considers cancer patients at the initial stage of their treatment, virtually all of them have primary tumor tissue available for detailed investigation. For the time being, all methods of liquid biopsy are based on the analysis of residual amounts of tumor fragments in body fluids. Therefore, it is hard to expect, that this indirect and potentially error-prone examination of single tumor cells or circulating tumor-specific molecules will indeed replace the direct tissue analysis in the near future. It is also essential to acknowledge, that the majority of actionable mutations demonstrate limited intratumoral heterogeneity; therefore, the analysis of a single tumor lump is usually sufficient for the choice of treatment and liquid biopsy is unlikely to have a high added value for the initial selection of optimal drugs (Jamal-Hanjani et al., 2017; Merker et al., 2018).

The situation becomes entirely different when the liquid biopsy is utilized during the treatment course. First of all, tumor response to the treatment cannot be always reliably assessed by the visualization of tumor lumps, especially given the fact that not all tumors are manifested by measurable tumor lesions. Marker response is an exceptionally valuable end-point for the evaluation of treatment efficacy. As already mentioned above, PSA and CA-125 are routine tools utilized in the management of prostate and ovarian cancer patients, respectively (Kobayashi et al., 2012; Hayes and Barry, 2014). While informative serum markers are available for the minority of human tumors, the use of blood-based mutation tests for the immediate assessment of treatment efficacy can potentially be applied to every cancer patient, as all malignant neoplasms contain a spectrum of somatic mutations. The utility of circulating tumor DNA for the control of surgical tumor eradication as well as the response to systemic treatment has already been exemplified in a number of studies (Tie et al., 2016; Abbosh et al., 2017; Garlan et al., 2017; Jamal-Hanjani et al., 2017; Goldberg et al., 2018; Lee et al., 2018). A similar approach can be applied for the early detection of tumor relapse.

The situation is getting more demanding while considering patients, whose tumors progressed after treatment. The phenotype of drug-resistant tumors may significantly evolve over time, and the choice of the right treatment strongly depends on the spectrum of newly acquired targets. For example, a novel lung cancer drug, osimertinib, has been intentionally developed to target non-small cell lung cancers, which progress after TKI therapy via acquisition of T790M mutation in *EGFR* gene (Lamb and Scott, 2017). The analysis of treatment-resistant tumors may require repetitive biopsies, which is not feasible if one considers a direct analysis of visceral, bone, or brain metastases. Furthermore, while intratumoral heterogeneity with regard to actionable mutations in treatment-naïve tumors is limited, the evolution of tumor-resistant lumps under the pressure of systemic treatment may utilize a multitude of alternative pathways even within the same patient (Suda et al., 2010; Pietrantonio et al., 2017). Liquid biopsy is expected to provide a more integral portrait of molecular events underlying tumor progression as compared to a single tissue-take (Gremel et al., 2016; Oxnard et al., 2016; Goodall et al., 2017).

There are hundreds of studies demonstrating the potential utility of the analysis of extracellular RNA for diagnosis of cancer disease (Xi et al., 2017; Zaporozhchenko et al., 2018). RNA is significantly less stable than DNA, due to its high vulnerability to RNA-ases as well to some other physical or chemical alterations. Nevertheless, RNA is relatively well preserved in various body fluids, as it is secreted as a part of microvesicles or lipoprotein complexes and therefore somehow protected from external hazards. Although the detection of extracellular RNA and the interpretation of obtained data is less straightforward as compared to mutation-based tests, there are some potential advantages of RNA testing. Some highly expressed extracellular RNA species are significantly more abundant than cell-free DNA, thus decreasing the requirements for the sensitivity of corresponding molecular tests. While DNA is released to the body fluids mainly due to cell death, the secretion of RNA is a physiological process; this is particularly relevant to some cancer types, e.g., adenocarcinomas of the lung, which are characterized by relatively low abundance of tumor-derived plasma DNA (Abbosh et al., 2017). There are some approaches, which allow to enrich the preparations of RNA by tissue-specific molecules, for example, by antibody-driven selection of particular category of exosomes. Some RNA species are present in increased amounts in body fluids, which are in close contact with the affected organ. For example, urine PCA3 RNA test is approved for the management of prostate cancer patients (Auprich et al., 2011). The development of other RNA-based assays is currently underway (Xi et al., 2017; Zaporozhchenko et al., 2018).

There is a hope that combined use of an array of DNA- and protein-based markers will result in a breakthrough in early cancer diagnosis and screening. The potential promise of these complex platforms has already been demonstrated in the study of early-stage cancers (Cohen et al., 2018). Nevertheless, many modern varieties of liquid biopsy still require proper clinical validation (Merker et al., 2018).

## The diagnosis of cancers of unknown primary site

Approximately 3–5% cancer patients with newly diagnosed metastatic disease have unknown organ or tissue origin for these metastases. In many instances, the inability to assign the right diagnosis is attributed purely to limitations in tumor visualization techniques. However, even autopsy fails to identify primary tumor in 15–45% patients with CUP (Pavlidis and Fizazi, 2009; Massard et al., 2011; Greco et al., 2012). This is compatible with the recent findings demonstrating that the spread of malignant cells may occur before the formation of the primary tumor lump (Harper et al., 2016; Hosseini et al., 2016). Furthermore, there are occasional examples of spontaneous regression of neoplastic lesions in the primary tumor site occurring simultaneously with the progression of distant metastases (Kamposioras et al., 2011). Despite all these limitations, correct determination of the organ/tissue origin for patients with CUP may result in appropriate treatment choice and improved outcome at least in a subset of cases (Hainsworth et al., 2013; Hainsworth and Greco, 2014; Varadhachary and Raber, 2014; Economopoulou et al., 2015).

The diagnostic approach to patients with CUP largely relies on common clinical sense. In particular, anatomic location of metastases, gender of the patients, knowledge on his/her smoking habits may significantly contribute to the diagnosis. IHC testing, which utilizes a spectrum of tissue-specific markers, is a gold standard for CUP clinical analysis. IHC has significant limitations. In particular, many expression-based markers are not sufficiently specific for a given tumor type. Some proteins are expressed at low levels and therefore cannot be detected by conventional antibody-based methods. The spectrum of diagnostic antibodies is restricted to the ones marketed by biotech companies. Finally, interpretation of IHC results is a subject of interlaboratory variations (Pavlidis and Fizazi, 2009; Massard et al., 2011; Economopoulou et al., 2015; Suspitsin et al., 2018).

DNA- and RNA-based tests may have some advantages as compared to IHC assays. In particular, some mutations are highly characteristic for cancers of a certain type. For example, presence of TKI-sensitizing somatic *EGFR* mutation in tumor tissue strongly favors the diagnosis of lung cancer; detection of *BRCA1/2* germ-line mutation in a female patient with adenocarcinoma of unknown primary site calls to consider breast or ovarian cancers as the most probable tumor variety. RNA expression markers could outperform some IHC tests, given that PCR-driven detection of RNA (cDNA) molecules does not have a sensitivity limit and can be applied to any expressed gene. In contrast to antibody production, the development of personalized PCR diagnostic tests does not require industrial facilities and can be done in any molecular genetic laboratory. Finally, PCR assays can be performed and interpreted in a semi-automated manner. Many studies demonstrate the utility of DNA/RNA analysis for determination of CUP origin (Talantov et al., 2006; Greco et al., 2010; Suspitsin et al., 2018). It is likely that NGS will provide more opportunities for CUP diagnosis in the near future (Varghese et al., 2017).

## Management of large amounts of biomedical data

Large-scale studies aimed at unbiased description of the entire spectrum of biological molecules in a given tissue or individual are often referred to as “omics” technologies. They include genomics (points mutations, copy number variations, single nucleotide polymorphisms), epigenomics (genome-wide analysis of DNA modifications, e.g., cytosine methylation), transcriptomics (spectrum and variants of expressed RNAs), proteomics (pattern of expressed proteins and their isoforms), metabolomics (analysis of various metabolites), etc. Sometimes these approaches result in the discovery of a single medically relevant marker, e.g., identification of causative gene or drug target (Jones et al., 2009; Iyer et al., 2012). However, the development of sophisticated molecular profiles is a more common output of the “omics” studies.

Many omics-derived classifiers provide an approach for semi-automated discrimination between different conditions, such as healthy status vs. malignant disease, high-risk vs. low-risk cancer, drug sensitivity vs. resistance, etc. Almost all high-throughput studies deal with datasets, in which the number of considered features significantly exceeds the number of analyzed cases. For example, while the expression microarrays are designed to simultaneously assess over twenty thousand genes, the number of included patients with different disease characteristics is usually limited to at best several hundreds of observations. In any event, this amount of data cannot be curated manually in a meaningful way, therefore the development of viable hypothesis and data interpretation are largely outsourced to computer intellect. There are many publicly available databases, including TCGA (https://cancergenome.nih.gov/), ICGC (http://icgc.org/), cBioPortal (http://www.cbioportal.org/), GEO (https://www.ncbi.nlm.nih.gov/geo/), COSMIC (https://cancer.sanger.ac.uk/cosmic), ExoCarta (http://www.exocarta.org/), MIRUMIR (http://www.chemoprofiling.org/cgi-bin/GEO/MIRUMIR/web_run_MIRUMIR.V1.pl), etc., which may serve as a data source for the development of new diagnostic tools. In addition, there are some research initiatives aimed at integration of high-throughput technologies with clinical trials (Pauli et al., 2017). Development of the artificial intelligence capable to adequately manage the flow of “big data” is an important challenge for translational research (Jagga and Gupta, 2015).

## Conclusions and perspectives

We are currently witnessing a revolution in medical research, which is attributed to the invention and rapidly increasing uptake of the next generation sequencing. NGS allows comprehensive description of germ-line DNA, analysis of somatic mutations and RNA profiles in naturally occurring tumors, systematic analysis of microbiomes, etc. There is an ongoing accumulation of data, which results in the identification of novel hereditary syndromes, molecular targets for cancer therapy, tumor-specific diagnostic markers, etc. We have to realize that the clinical integration of relatively simple and straightforward assays, like *BRCA1/2* analysis or *EGFR* mutation testing, took several years each, with many issues remaining unresolved even for the time being. It is difficult to foresee, how practical medicine will manage an overwhelming flow of novel candidate markers, given that they are represented by a multitude of rare and diverse molecular events and therefore cannot be clinically validated on the individual basis. These advances may need to be considered while discussing the standards of clinical research, data dissemination and interaction between clinical and laboratory specialists.

## Author contributions

All authors listed have made a substantial, direct and intellectual contribution to the work, and approved it for publication.

### Conflict of interest statement

The authors declare that the research was conducted in the absence of any commercial or financial relationships that could be construed as a potential conflict of interest.
