# Supplementary material for: Molecular Diagnostics in Clinical Oncology
Source: Front Mol Biosci. 2018 Aug 27;5:76. doi: 10.3389/fmolb.2018.00076 (PMC6119963; doi:10.3389/fmolb.2018.00076)
Supplement: Supplementary file 1 [file Data_Sheet_1.pdf]

Supplementary material

Molecular diagnostics in clinical oncology

Anna P. Sokolenko*, Evgeny N. Imyanitov*

***Correspondence:** Anna P. Sokolenko, annasokolenko@mail.ru; Evgeny N. Imyanitov, evgeny@imyanitov.spb.ru

Literature search criteria

Search for recent reviews on predictive markers (papers devoted to specific cancer type are excluded)

review [ptyp] AND (cancer [title] OR carcinoma [title] OR tumor [title] OR tumors [title] OR tumour [title] OR tumours [title] OR malignancy [title] OR malignancies [title] OR neoplasm [title] OR neoplasms [title]) AND (predict* OR efficacy OR success OR response OR effect OR customized OR individualized OR survival) AND (therapy OR treatment) AND (2017 [pdat] OR 2018 [pdat]) AND english [lang] AND (expression OR molecular OR gene OR polymorphism OR allele OR SNP OR genetic OR profile OR profiling OR array OR microarray OR marker OR mutation OR rearrangement) NOT (breast [title] OR gastr* [title] OR stoma* [title] OR colo* [title] OR pancrea* [title] OR lymph* [title] OR prostat* [title] OR bladder [title] OR melanoma [title] OR head [title] OR sarcom* [title] OR neuro* [title] OR esophageal [title] OR esophagus [title] OR ovar* [title] OR lung [title] OR renal [title] OR kidney [title] OR cholangio* [title] OR biliary [title] OR brain [title] OR glio* [title] OR leuke* [title] OR myeloma [title] OR liver [title] OR hepa* [title] OR urolo* [title])

Search for recent reviews on hereditary cancers (papers devoted to specific cancer type are excluded)

review [ptyp] AND (cancer [title] OR carcinoma [title] OR tumor [title] OR tumors [title] OR tumour [title] OR tumours [title] OR malignancy [title] OR malignancies [title] OR neoplasm [title] OR neoplasms [title]) AND (heredit* [title] OR famil* [title] OR predispos* [title]) AND (2016 [pdat] OR 2017 [pdat] OR 2018 [pdat]) AND english [lang] AND (expression OR molecular OR gene OR polymorphism OR allele OR SNP OR genetic OR profile OR profiling OR array OR microarray OR marker OR mutation OR rearrangement) NOT (breast [title] OR gastr* [title] OR stoma* [title] OR colo* [title] OR pancrea* [title] OR lymph* [title] OR prostat* [title] OR bladder [title] OR melanoma [title] OR head [title] OR sarcom* [title] OR neuro* [title] OR esophageal [title] OR esophagus [title] OR ovar* [title] OR lung [title] OR renal [title] OR kidney [title] OR cholangio* [title] OR biliary [title] OR brain [title] OR glio* [title] OR leuke* [title] OR myeloma [title] OR liver [title] OR hepa* [title] OR urolo* [title])

Search for recent reviews on liquid biopsy (papers devoted to specific cancer type are excluded)

review [ptyp] AND (cancer [title] OR carcinoma [title] OR tumor [title] OR tumors [title] OR tumour [title] OR tumours [title] OR malignancy [title] OR malignancies [title] OR neoplasm [title] OR neoplasms [title]) AND (“liquid biops*” [title] OR “circulating DNA” OR “circulating microRNA*” OR “circulating nucleic”) AND (2017 [pdat] OR 2018 [pdat]) AND english [lang] NOT (breast [title] OR gastr* [title] OR stoma* [title] OR colo* [title] OR pancrea* [title] OR lymph* [title] OR prostat* [title] OR bladder [title] OR melanoma [title] OR head [title] OR sarcom* [title] OR neuro* [title] OR esophageal [title] OR esophagus [title] OR ovar* [title] OR lung [title] OR renal [title] OR kidney [title] OR cholangio* [title] OR biliary [title] OR brain [title] OR glio* [title] OR leuke* [title] OR myeloma [title] OR liver [title] OR hepa* [title] OR urolo* [title] OR endometr* OR thyroid*)

Search for papers on cancers of unknown primary site

((cancer AND CUP) OR (adenocarcinoma AND CUP) OR “cancer of unknown primary”) AND (PCR OR RNA OR clinical trial [ptyp] OR mutation OR review [ptyp]) AND English [lang]

Search for recent studies on molecular diagnostics (only top journal are considered; reviews are excluded)

(cancer [title] OR carcinoma [title] OR tumor [title] OR tumors [title] OR tumour [title] OR tumours [title] OR malignancy [title] OR malignancies [title] OR neoplasm [title] OR neoplasms [title]) AND (predict* OR efficacy OR success OR response OR effect OR customized OR individualized OR survival) AND (therapy OR treatment) AND (2016 [pdat] OR 2017 [pdat] OR 2018 [pdat]) AND english [lang] AND (expression OR molecular OR gene OR polymorphism OR allele OR SNP OR genetic OR profile OR profiling OR profiling OR array OR microarray OR marker OR mutation OR sequencing OR heredit* [title] OR familial [title] OR predispos* [title] OR “liquid biops*” [title] OR “circulating DNA” OR “circulating microRNA” OR “circulating nucleic” OR “plasma DNA” OR “plasma microRNA”) AND (Cancer Res [jour] OR Clin Cancer Res [jour] OR Science [jour] OR Nature [jour] OR Nat Med [jour] OR Nat Genet [jour] OR Lancet [jour] OR Lancet Oncol [jour] OR Proc Natl Acad Sci USA [jour] OR N Engl J Med [jour] OR Am J Hum Genet [jour] OR J Natl Cancer Inst [jour] OR Ann Oncol [jour] OR J Clin Oncol [jour] OR BMJ [jour] OR Sci Transl Med [jour] OR Cancer Discov [jour] OR JAMA Oncol [jour]) NOT review [ptyp]
